# Repetitive transcranial magnetic stimulation can improve the fixation of eyes rather than the fixation preference in children with autism spectrum disorder

**DOI:** 10.3389/fnins.2023.1188648

**Published:** 2023-07-20

**Authors:** Li Tian, Shuai Ma, Yin Li, Meng-fei Zhao, Chang Xu, Chen Wang, Xin Zhang, Lei Gao

**Affiliations:** ^1^Tianjin Anding Hospital, Tianjin, China; ^2^Department of Maternal, Child and Adolescent Health, School of Public Health, Tianjin Medical University, Tianjin, China

**Keywords:** autism spectrum disorders (ASD), repetitive transcranial magnetic stimulation (rTMS), dorsolateral prefrontal cortex (DLPFC), mediation analysis, eye tracking (ET)

## Abstract

**Background:**

Transcranial magnetic stimulation (TMS) has been introduced into the intervention of autism spectrum disorders (ASD) as a possible new therapeutic option for modifying pathological neuroplasticity. However, the stimulating protocols of rTMS for ASD have not been approved unanimously, which affects the clinical popularization and application of rTMS. In addition, there is little research on the improvement of social processing of autistic children by rTMS.

**Methods:**

We explored the clinical efficacy of rTMS and improvement of face processing with the protocol of left high-frequency and right low-frequency on bilateral dorsolateral prefrontal cortex (DLPFC), with a sample of 45 ASD participants aged 2–18.

**Results:**

Our results showed that both the score on the Childhood Autism Rating Scale (CARS) and the fixations on the eyes of the human faces improved by two-session rTMS intervention, except for the percentage of eyes fixation. The mediation analysis indicated the item of “Adaptation to Change” of CARS mediated dominantly the improvement of eye-gaze behavior of ASD participants by rTMS.

**Conclusion:**

Our study revealed the mechanism of rTMS in improving the eye-gaze behavior of the autism population, deepened the understanding of the function of rTMS in treating autistic social disorders, and provided a reference for combined treatment for ASD.

## 1. Introduction

Autism spectrum disorder (ASD) is a lifelong neurodevelopmental disorder, which occurs in early childhood and is characterized by social disorder, language communication disorder, limited interest range and/or repetitive behaviors (Lord et al., 2018). The prevalence of ASD continues to rise worldwide (Chiarotti and Venerosi, 2020). For example, according to the Autism and Developmental Disabilities Monitoring (ADDM) Network in the United States, the estimated prevalence of ASD increased from 6.7 (one in 150) per 1,000 children aged 8 years at ADDM Network sites in 2000 and 2002 to 23.0 (one in 44) in 2018 (Maenner et al., 2021). In China, the prevalence of ASD is estimated to reach 1% (Sun et al., 2019). Over 70% of individuals with ASD require lifelong care and rehabilitation (Kong et al., 2017), and the average lifetime cost of each ASD individual is approximately 3.6 million US dollars (Cakir et al., 2020), bringing a substantial economic burden to their families and society. Therefore, finding a more safe and effective intervention method has become a significant problem to be solved urgently in the research field of ASD.

At present, most therapeutic interventions in ASD only provide symptomatic treatment, and the outcomes of the intervention are judged by subjective endpoints (such as behavioral evaluation) which together with the high heterogeneity of ASD account for the wide variability in the effectiveness of treatments (Casanova et al., 2020b). Transcranial magnetic stimulation (TMS) is one of the first treatments that target a putative core pathological feature of ASD, specifically the cortical inhibitory imbalance that alters gamma frequency synchronization (Sokhadze et al., 2009; McNally and McCarley, 2016). Previous studies showed that low-frequency TMS over the dorsolateral prefrontal cortex (DLPFC) of individuals with ASD decreases the power of gamma activity and increases the difference between gamma responses to target and non-target stimulation (Casanova et al., 2021), which improves executive function skills related to self-monitoring behavior and the ability to take corrective measures (Casanova et al., 2020b). These improvements are not only reflected in the reduction of stimulus-bound behaviors (Casanova et al., 2020b), but also shown as diminished sympathetic arousal (Wang et al., 2016). Moreover, the improvement also presents a dose-response relationship, i.e., the more number of TMS sessions, the more improvement in ASD symptoms (Oliveira et al., 2018). Although TMS has shown some positive effects in treating ASD, there are still some key problems have not been solved, such as the stimulating protocols and the stimulating sites, which affect the clinical popularization and application of rTMS (Krishnan et al., 2015). For example, Baruth et al. (2010) used low-frequency rTMS to stimulate the DLPFC of ASD patients and found that irritability and repetitive behavior could be improved (Baruth et al., 2010); while Enticott et al. (2014) found that they improved the social disorder and anxiety of ASD patients by stimulating bilateral dorsomedial prefrontal cortex with high-frequency rTMS (Enticott et al., 2014). Our previous study has shown that the high-frequency rTMS on left DLPFC and low-frequency on right DLPFC can improve ASD symptoms as well as sleep disturbances (Gao et al., 2021). The possible therapeutic mechanism of that rTMS protocol includes: (1) The high-frequency rTMS stimulation of the left DLPFC could cause long-term potentiation (LTP) of synaptic transmission in the stimulation area (Rajji et al., 2013), and LTP could spread to the cortex and sub-cortical neural network (Fox et al., 2012; Shafi et al., 2012), which led to the enhancement of excitability of mirror neuron system (MNS) system in ASD patients, to improve the understanding of social environment in ASD patients, enhance the ability of imitation (Yang et al., 2019), by which to improve the social function. (2) the low-frequency rTMS over the right DLPFC could improve the abnormal brain wave activity patterns in the gamma bandwidth in ASD patients (Casanova et al., 2020a,^2021^), such as normalizing gamma oscillation abnormalities (Baruth et al., 2010; Casanova et al., 2020a), executive functions (Ameis et al., 2017; Barahona-Corrêa et al., 2018; Khaleghi et al., 2020), and repetitive behaviors (Oberman et al., 2016; Khaleghi et al., 2020) in ASD individuals. Thus, the high-frequency rTMS on left DLPFC and low-frequency on right DLPFC seemed quite promising in ASD treatment.

Meanwhile, there are also few studies on the improvement of facial processing features of children with ASD. As we know, the abnormal processing of human faces in ASD population is considered to be the most significant social defect feature (Bird et al., 2006). On the one hand, ASD individuals are shown to avoid others’ eye contact in social situations (Yi et al., 2013; Madipakkam et al., 2017); on the other hand, they lack attentional preference for faces [that is, the attention preference for human faces, relative to non-face stimuli, presented by typically developed children at birth (Penton et al., 2022)] in the environment (Behrmann et al., 2006; Kikuchi et al., 2009; Chawarska et al., 2010). Eye-movement studies have also shown that young children with ASD under the age of 3 years exhibit a range of socio-visual attention deficits, such as reduced gaze to the eyes (Jones et al., 2008; Jones and Klin, 2013) and face area (Shic et al., 2011), which have been regarded as biomarkers of early social development abnormalities in ASD individuals (Pierce et al., 2016). Neuroimaging studies also indicated that ASD individuals’ defects in face processing may be related to the abnormal activation of DLPFC (Richey et al., 2022). The exploration of improvement of abnormal face processing by rTMS on DLPFC of ASD participants will play a positive role in deepening the understanding of neural mechanisms of social processing of ASD participants and promoting the better application of rTMS in the clinical intervention of ASD.

Therefore, we explored the clinical efficacy of rTMS and improvement of face processing with the protocol of left high-frequency and right low-frequency on bilateral DLPFC. For the face processing, the preferential looking paradigm was used, with the area of interest (AOI) of the eyes and the whole face, to check the fixations on eyes before and after the intervention. To further explore the possible mechanism of rTMS on face processing, we also planned to make the mediation analysis, with the score of the Childhood Autism Rating Scale (CARS) as the mediator. We hypothesized that rTMS with above protocol could effectively improve the facial fixation of ASD children, not only on the eyes of faces, but also the attentional preference for eyes (Richey et al., 2022).

## 2. Methodology

### 2.1. Subjects

We mainly carried out this study in Tianjin Anding (psychiatric) Hospital from October 2018 to October 2021 and released the recruitment information to hospitalized patients or outpatients with ASD and evaluated the subjects who wanted to participate in the development lab of Tianjin Medical University. The eligibility criteria included: (1) it met the diagnostic criteria of ASD in the fifth edition of the American Diagnostic and Statistical Manual of Mental Disorders (DSM-V); (2) age of 2–18 years old; (3) no medication during the rTMS intervention; (4) right-handed; (5) the total score of CARS in the baseline ≥30 (Schopler et al., 1980). The exclusion criteria were (Sokhadze et al., 2012; Tian et al., 2021): (1) contraindications to rTMS, such as metal or electronic instruments near the coil stimulation site; participants with a history of epilepsy (excluding epilepsy according to their electroencephalogram and medical record); participants with a history of brain trauma, brain tumors, and other diseases; participants with severe or recent heart disease; or other major physical illness. (2) Diagnosis of other mental illnesses (e.g., attention-deficit hyperactivity disorder, schizophrenia and depression). (3) Other neurodevelopmental disorders, genetic metabolic diseases, or severe neurological diseases. (4) Participants who could not cooperate with the eye movement experiment.

The study was conducted under the Code of Ethics of the World Medical Association (Declaration of Helsinki). Also, the study complied with all relevant national regulations and institutional policies and had been approved by the Medical Ethics Committee of Tianjin Medical University. Participants and their parents (or legal guardians) obtained all information about the research, including the purpose, requirements, responsibilities, compensation, risks, benefits, and alternatives. All questions were answered before asking for the consent signature.

### 2.2. TMS procedure

A trained electrophysiologist delivered rTMS stimulation over the cortical area controlling the contralateral First Dorsal Interosseous (FDI) using a Magnetic Field Stimulator (CCY-1, YIRUIDE Medical Corporation, Wuhan, China) to detect resting motor threshold (MT). The MT was determined for each hemisphere in all individuals by gradually increasing the output of the machine by 5% until a 5 mV deflection or a visible twitch in the FDI muscle was identified in 2 out of 3 trials (Sokhadze et al., 2012). Electromyographic (EMG) responses were monitored continuously from the hand contralateral to the stimulated hemisphere using the MEP module in Magnetic Stimulator (YIRUIDE Medical Corporation, Wuhan, China). Subjects were familiarized with the laboratory and procedure before the first TMS session.

In this study, rTMS was selected to stimulate left DLPFC with high frequency (10 Hz) and right DLPFC with low frequency (1 Hz) based on the evidence-based basis proposed by the European Union of Neurological Societies (Brainin et al., 2004), and the electrode positioning cap was used for accurate positioning, as shown in Supplementary Figure S1 in Attachment (the electrode positioning cap used in the actual intervention was a children’s model). Specific parameters are as follows: stimulation frequency of right dorsolateral prefrontal lobe is 1 Hz, stimulation time is 32 s, stimulation number is 32, intermittent time is 1 s, repetition number is 28, the stimulation intensity is 25% RMT; stimulation frequency of left dorsolateral prefrontal lobe is 10 Hz, stimulation time is 3.2 s, stimulation number is 32. Intermittent time is 10 s, repetition number is 45. Stimulation intensity is 25% RMT. The intervention time of rTMS was 5 times/week, and every 4 weeks was a course of intervention. The circumstance and posture of participants at the time of rTMS intervention are shown in Supplementary Figure S2 Attachment.

### 2.3. Eye tracking procedure

**FIGURE 1:**
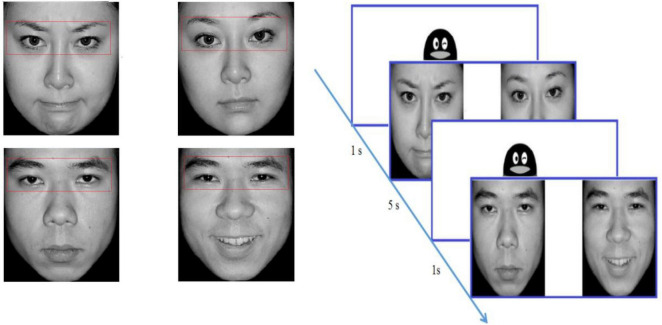
The examples, definitions of AOI and flowchart in the visual preference experiments.

The stimuli were selected from the Chinese Affective Picture System (Bai et al., 2005) and consisted of 48 different pictures (48 emotional pictures and 48 neutral pictures). Each picture included two black-and-white photographs of the same person with varied emotional valence (positive/negative + neutral), and the two photographs were equal in size and symmetrical in position. When appearing together, the two photographs were located approximately 5° of visual angle away from each other. The size of every picture was 720 × 480 pixels, subtending a visual angle of 13.78°in height by 7°in width. There were three factors in this study, including the gender of faces (male, female), the left or right visual field where the emotional pictures were presented (LVF, RVF), and the picture valence (positive, negative). There was one block for each condition and 6 trials in each block. Examples of face stimuli are presented in Figure 1.

We used a Tobii TX300 eye tracker and the Tobii Studio software to present the stimuli, record eye movements, and analyze the gazing behavior of the participants. The fixation was defined as continuous gazing for more than 80 ms within a 1 degree of visual angle or 30 pixels. The experiment took place in a controlled environment (illumination, temperature, etc.) in the development laboratory of the Department of Maternal, Child and Adolescent Health at Tianjin Medical University.

Participants were instructed to look at the pictures on the monitor after completing 9-point calibration. The pictures were presented in randomized order for a duration of 5 s at a sampling rate of 120 Hz by using Tobii Studio 3.0 Eye Tracking Software. Between two trials, an image of a cartoon penguin over a white background was presented at the center of the screen for 1 s (see the flow chart in Figure 1). The subject was not required to respond when viewing the picture. For uncooperative subjects, their caregivers could stay, but they are not allowed to watch screen. For the uncooperative subjects, their caregivers could stay but are not allowed to view the screen.

We used the eyes of the left and right faces as the Area of Interest (AOI). The eye movement parameters analyzed in this study included: ① fixation count (FC), which refers to the number of times the participant fixated on an AOI; ② total fixation duration (TFD): the sum of the duration of the subject’s fixation in the AOI. To show the eye preference, we calculated the percentage of eyes fixation, i.e., TFD of eyes in one certain face was divided by the TFD of that whole face to derive the proportion of time spent on eyes (i.e., “% eyes”).

### 2.4. Clinical assessments

We evaluated the symptoms of ASD with CARS. The CARS consists of 14 domains assessing behaviors associated with autism, with a 15th domain rating general impressions of autism. Each field has a scale of one to four. Higher scores indicate a higher level of impairment. Total scores can range from 15 to 60. Scores below 30 mean that the individual is in the non-autistic range, a score between 30 and 36.5 indicates mild to moderate autism, and scores between 37 and 60 indicate severe autism (Chlebowski et al., 2010).

### 2.5. Statistical analysis

We used EpiData to build the database and SPSS 22.0 to make statistical analysis. We used the repeated Measures Analysis of Variance (RMANOVA) to compare the effect of rTMS, with FC, TFD and the number of pictures that ASD participants neglected (no fixation on the whole picture) as dependent measures, respectively. As to eye preference, and *location* (left visual field vs. right visual field), *gender* (male face vs. female face) and *emotion* (positive vs. negative face) as within-subject factors, and *time* (before vs. One-session rTMS vs. Two-session rTMS) as the between-subjects factors. For the mediation analysis, the model of Bayesian mediation analysis was created with time as the independent variable (Time = 0, 1, 2 as pre, post rTMS), the score of CARS, including the total score and scores of the subscales as the mediator, respectively, and the FC or TFD as the dependent variable by using the procedure of MCMC of SAS 9.4 (Enders et al., 2013).

### 2.6. Community involvement

A total of 45 autistic children were involved in this study. Their parents also provided help for the smooth implementation of this study. The publicity of The China Disabled Persons Federation (CDPF) of Tianjin also helped the smooth implementation of this study.

## 3. Results

### 3.1. The demographics of the participants

In the study, 45 ASD participants completed at least two intervention sessions (4 weeks per session) and completed the assessment. Among them, 36 completed two sessions, 8 completed three sessions, and one subject completed four sessions. There were 37 males (77.8%) and 8 females (22.2%), with an average age of 8.802 ± 4.171 years; and the average score of CARS was 36.95 ± 6.82 (see Table 1).

**TABLE 1:** The demographics of the ASD participants (*n* = 45).

		*n* (%)
Gender		
	Male	37 (77.8%)
	Female	8 (22.2%)
Age		
	2∼	12 (26.7%)
	6∼	25 (55.5%)
	12∼18	8 (17.8%)
CARS		
	30∼36	29 (64.4%)
	≥36	16 (35.6%)

### 3.2. The results of fixation on eyes after rTMS

**FIGURE 2:**
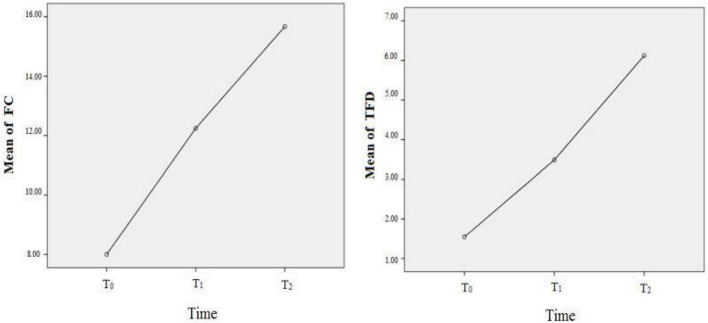
The improvement of fixation on eyes before, after rTMS. T_0_ = before rTMS intervention; T_1_ = afterone-session rTMS intervention; T_2_ = after two-session rTMS intervention.

For the fixation on the facial eyes, the FC, and TFD as the independent variables, respectively, the main effect of *time* was statistically significant (*F_*FC*_* = 6.147, *P* = 0.003; *F_*TFD*_* = 10.159, *P* < 0.001), which meant that both FC and TFD were significantly improved compared to the baseline (before rTMS). However, as to the number of pictures that ASD participants neglected, the main effect of *time* was not statistically significant. That is to say, even after rTMS intervention, some ASD participants still missed some facial images. For further comparisons of different times, only the comparison between baseline and two-session rTMS was statistically significant for FC; but for TFD, the comparisons were statistically significant except for the comparison between one-session rTMS and two-session rTMS, see Figure 2 and Table 2. The heat map of facial gaze before and after the intervention is shown in Supplementary Figure S3 in Attachment.

**TABLE 2:** The comparisons of FC and TFD among different time.

Parameters	(I) Time	(J) Time	Δ (I-J)	S.E	*P*	95% CI	
						Lower	Upper
**FC**	T_0_	T_1_	-4.244	2.091	0.131	-9.339	0.851
		T_2_	-7.667	2.358	0.005	-13.407	-1.926
	T_1_	T_0_	4.244	2.091	0.131	-0.851	9.339
		T_2_	-3.422	2.113	0.293	-8.573	1.729
	T_2_	T_0_	7.667	2.358	0.005	1.926	13.407
		T_1_	3.422	2.113	0.293	-1.729	8.573
TFD	T_0_	T_1_	-1.941	0.753	0.037	-3.790	-0.093
		T_2_	-4.576	1.041	<0.001	-7.145	-2.008
	T_1_	T_0_	1.941	0.753	0.037	0.093	3.790
		T_2_	-2.635	1.210	0.094	-5.588	0.318
	T_2_	T_0_	4.576	1.041	<0.001	2.008	7.145
		T_1_	2.635	1.210	0.094	-0.318	5.588

FC = fixation count; TFD = total fixation duration; T_0_ = before rTMS intervention; T_1_ = after one-session rTMS intervention; T_2_ = after two-session rTMS intervention.

### 3.3. The results of the percentage of eyes fixation after rTMS

Although after rTMS intervention, subjects with ASD showed an increase in eye fixation, the percentage of eyes fixation remained unchanged. The main effect of *time* remained statistically insignificant for FC (*F* = 0.563, *P* = 0.571) and TFD (*F* = 0.022, *P* = 0.978). None of the interactions of gender * time, emotion * time and location * time was statistically significant.

### 3.4. The improvement of CARS by rTMS

The total score of CARS showed constant improvement by rTMS, from 36.95 ± 6.82 (baseline) to 33.178 ± 5.921 (after one session), then to 29.756 ± 5.974 (after two sessions). By one-way ANOVA, the change of CARS score was statistically significant (*F* = 21.203, *P* < 0.001), the further comparisons showed that the improvement of different times was all statistically significant, T_0_ vs. T_1_: *t* = 3.765, *P* = 0.001; T_1_ vs. T_2_: *t* = 2.729, *P* = 0.023; T_0_ vs. T_2_: *t* = 6.463, *P* < 0.001.

### 3.5. The results of mediation analysis

**FIGURE 3:**
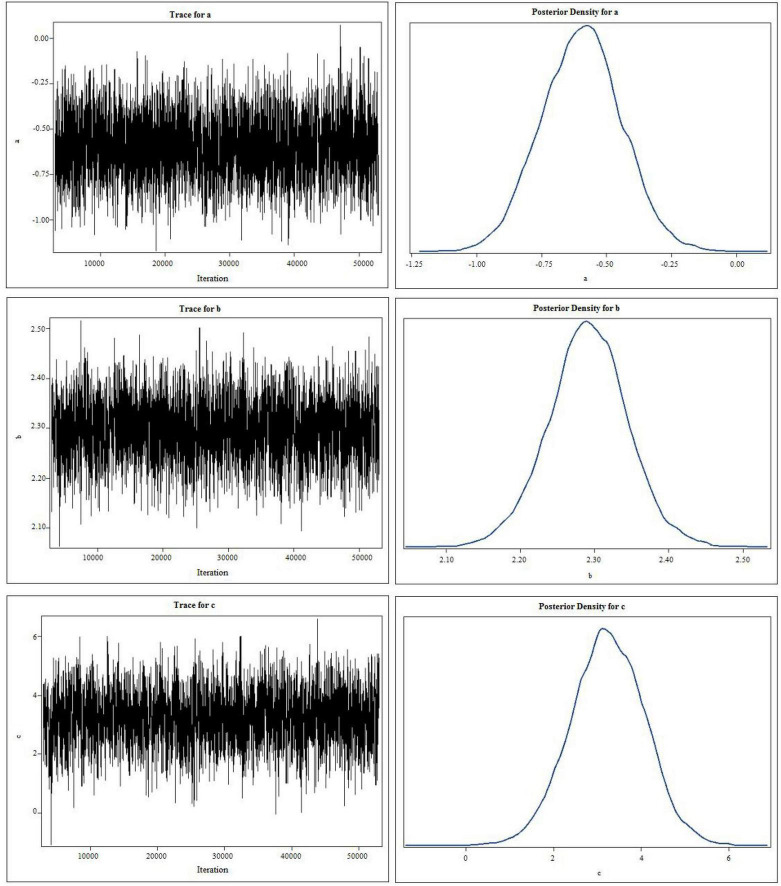
The trace plots and kernel density plots of the posterior distribution for the parameters. “c” represents the total effect of independent variable (X) on the dependent variable (Y), “b” measures the relation between the mediator M and the dependent variable Y adjusted for the independent variable X, and “a” measures the relation between X and M.

First, we performed a Bayesian mediation analysis with a total score of CARS mediating the relationship between rTMS intervention and the change of TFD (the score of TFD after two sessions minus the baseline). However, the 95% central credibility interval was [−0.731, 0.276], which contained 0 and meant the mediated effect is not statistically significant. Then the score of each item (such as Relating to People, Imitation, Emotional Response, Body Use, Object Use, Adaptation to Change, Visual Response, Listening Response, Taste, Smell, and Touch Response and Use, Fear or Nervousness, Verbal Communication, Non-verbal Communication, Activity Level, Level and Consistency of Intellectual Response and General Impressions) was taken as the mediator, respectively, and only Item 6 (Adaptation to Change) was the mediator, the rest was rejected due to the poor convergence of the Markov chain or the containment of 0 in the 95% central credibility interval, which meant the mediated effect is not statistically significant. As shown in Figure 3, the trace plots indicated the good mixing for parameters, and chains that mix well tend to converge sooner, as well as the kernel density plots of the posterior distribution for the given parameter. The posterior mean of the mediated effect of rTMS intervention through the score of *Adaptation to Change* on change in the TFD was αβ = −1.735 ± 0.515 with a 95% central credibility interval [−2.950, −1.231]. Given that zero was not between the two credibility limits, the mediated effect of SSP was statistically significant. Further calculation (αβ/c × 100%) reflected the mediation effect was 83.24%, meaning that 83.24% of the total effect between rTMS intervention and change in the TFD was mediated by the score of *Adaptation to Change* (see Table 3). This indicated that rTMS intervention improved the fixation on eyes mainly by promoting their adaptation to environmental change.

**TABLE 3:** Parameter summary of Bayesian mediation analysis with SSP as mediator.

Parameter	*N*	Mean	Stand deviation	95% C I of HPD	
				Lower	Upper
α	50,000	-0.616	0.103	-0.841	-0.458
β	50,000	3.066	0.422	2.359	3.635
*c*	50,000	2.295	0.352	1.659	3.436
*h*	50,000	-0.769	0.108	-0.976	-0.566
αβ	50,000	-1.735	0.595	-2.950	-1.231

“c” represents the total effect of independent variable (X) on the dependent variable (Y), “αβ” represents the effect of X on Y adjusted for the effect of the mediator M, “β” measures the relation between the mediator M and the dependent variable Y adjusted for the independent variable X, and “α” measures the relation between X and M.

## 4. Discussion

We mainly examined the efficacy of rTMS protocol (high frequency on left + low frequency on right) on bilateral DLPFC for both the clinical symptoms (score of CARS) and facial fixation in ASD participants. In the current study, we found that after rTMS intervention, CARS scores significantly decreased, and the decline of CARS score correlated with the extension of the treating sessions, showing a significant dose-response relationship, which was consistent with Casanova’s review (Casanova et al., 2020b). Also, our results showed that the ASD participants fixated more on the eyes of the human faces after two sessions of rTMS, including FC and TFD. Li (2021) used the excitatory (50 hz) intermittent Theta-Burst Stimulation (iTBS) to intervene the right posterior superior temporal sulcus of autistic individuals, after 28-days intervention, the ASD participants showed an increased fixation on the eyes of the emotional faces, which is consistent with our results (Li, 2021).

Although our results showed that rTMS intervention could improve the eye-fixation of ASD subjects, they did not improve the percentage of eye fixation, i.e., the ASD participants failed to use eye as the most important area (eye-gaze preference) as their TD peers. The lack of eye-gaze preference indicates the insufficiency of using eye information in face processing, which is also one of the characteristics of autistic subjects (Pelphrey et al., 2002; Spezio et al., 2007). These abnormalities are usually explained as the result of both congenital injury of specialized nervous systems and the secondary result of reduced social interest (Sasson, 2006). Face processing is an emergent and developmental skill that is greatly influenced by early experience with faces (Ellis, 1992; Taylor et al., 2004; Pascalis et al., 2011). ASD individuals may possess central nervous system irregularities that fail to attribute special status to faces (Sauer et al., 2021), which attenuates the visual input required for the development of neural regions specialized for face processing (Sasson, 2006; Campatelli et al., 2013). Even if rTMS treatment could improve the central nervous system abnormalities and the decreased social interest of autistic individuals, it is unlikely to make up for their lack of early face processing experience in a limited period. Therefore, it is necessary to provide other measures, such as social skills training, to promote their social impairments besides rTMS intervention. Meanwhile, our results suggest the value of early application of rTMS intervention in autistic populations, especially in the critical period of their social development, so that their social development can be corrected as early as possible, and obtain as many social skills as possible. Further, the results of mediation analysis showed that the improvement of adaptability of ASD children to environmental changes played a critical role in the increment of fixation on the eyes. However, we failed to find any literature on this issue. As we know, changes, especially unexpected changes, can be extremely stressful for children with National Institute for Health and Care Excellence (2021). When change occurs, children with ASD may feel anxious and respond in a variety of ways, including exhibiting withdrawal, repetitive behaviors, tantrums, or even aggression (Lau et al., 2020). And the rTMS protocol (left high-frequency and right low-frequency on bilateral DLPFC) in our study has been proven effective in treating anxiety (Chen et al., 2019; Abdelrahman et al., 2021). Thus, we speculate that relieving anxiety will play a role in increasing the eye-gaze behavior of autistic individuals, because anxiety symptoms are associated with eye-gaze avoidance (Staab, 2014; Michalska et al., 2017). We will further explore whether the anxiety symptoms play a role in the visual avoidance of autism in the future, by using the anxiety scale (such as the self-rating anxiety scale) and neurophysiological indicators (such as pupillary response, heartbeat, skin resistance, etc.) among high-function autism patients, or we can study the eye-gaze behavior of autism patients through the anti-anxiety medications. Our results have deepened the understanding of the function of rTMS in treating the social disorders of the autism population and provided a reference for combined treatment.

There are some limitations in our study, for example, the utilization of static facial images with relatively low ecological validity, and the sample size in the current study was relatively small. We plan to conduct face-to-face interaction in future research to improve ecological validity. Only one rTMS protocol was used without others, such as different frequencies on bilateral DLPFC or the parietal lobe (Sokhadze et al., 2010; Yang et al., 2019), due to the limitation of time and research funds.

## Data availability statement

The datasets presented in this article are not readily available because their containing information in the data probably compromise the privacy of research participants. Requests to access the datasets should be directed to LG, gaolei98@tmu.edu.cn.

## Ethics statement

The studies involving human participants were reviewed and approved by the Medical Ethics Committee of Tianjin Medical University. Written informed consent was obtained from the individual(s) or the participants’ legal guardian/next of kin for the publication of any identifiable images or data included in this article.

## Author contributions

LT, SM, and LG contributed to the conception and design of the study and wrote the first draft of the manuscript. LT, SM, YL, M-FZ, CX, CW, and XZ contributed to the acquisition, analysis, or interpretation of data. LG performed the statistical analysis. LG and XZ obtained funding. All authors contributed to the article and approved the submitted version.
