# Supplementary material for: Repetitive transcranial magnetic stimulation can improve the fixation of eyes rather than the fixation preference in children with autism spectrum disorder
Source: Front Neurosci. 2023 Jul 20;17:1188648. doi: 10.3389/fnins.2023.1188648 (PMC10400712; doi:10.3389/fnins.2023.1188648)
Supplement: Supplementary file 1 [file Table_1.DOCX]

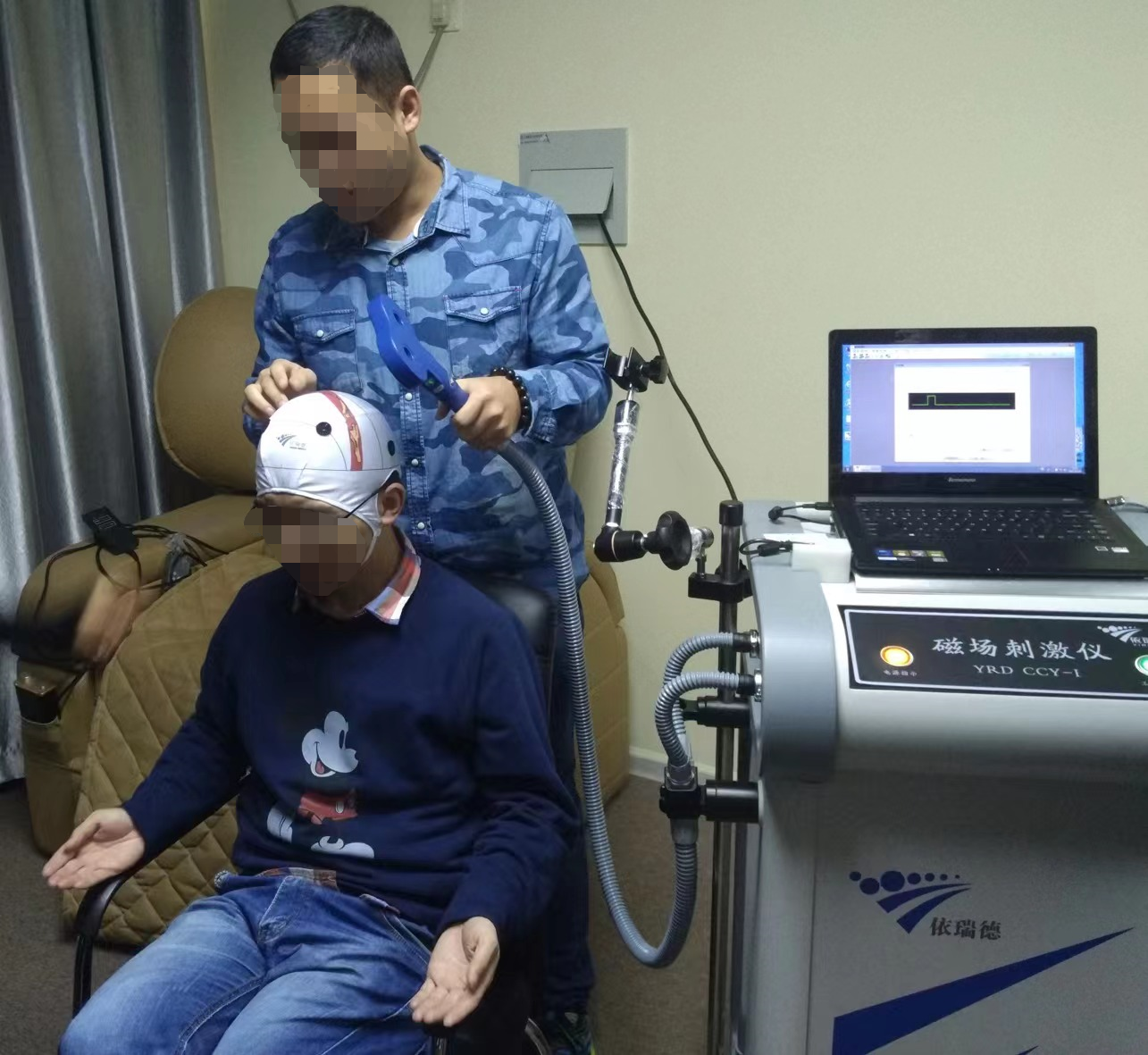


Figure S1 The schematic diagram of the electrode positioning cap

*Note*:The electrode positioning cap used in the actual intervention was a children's model


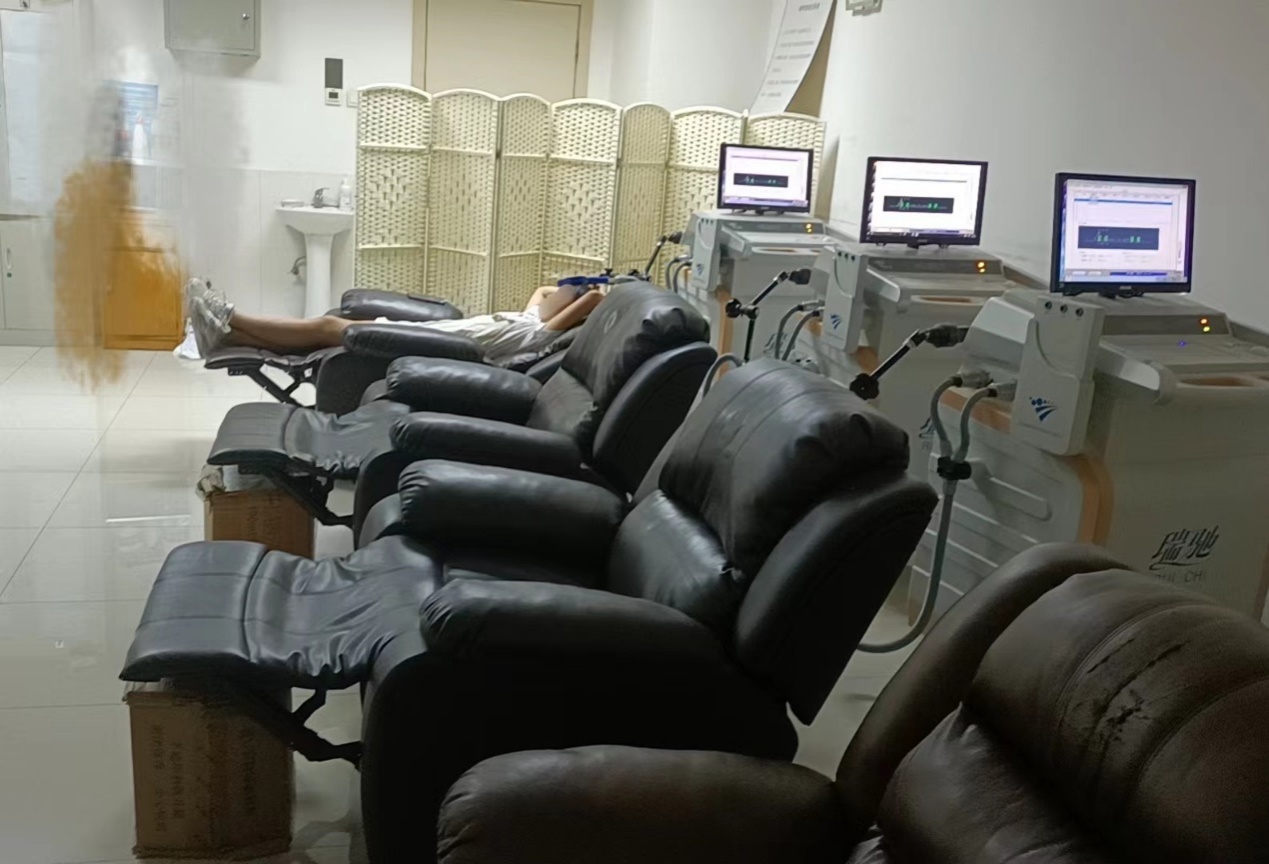


Figure S2 The circumstance and posture of participants at the time of rTMS intervention


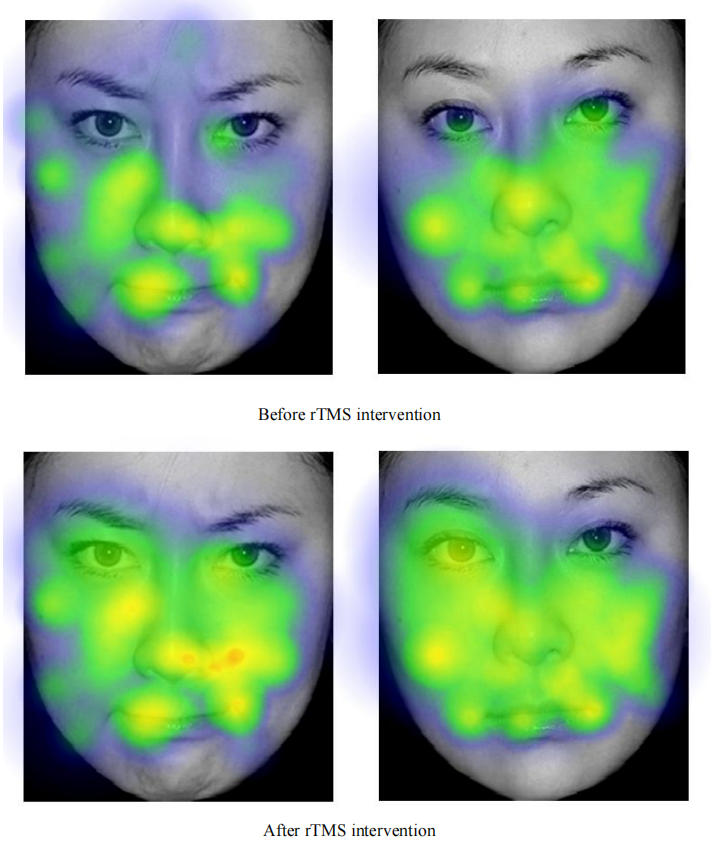


Figure S3 The heat map of facial gaze before and after the intervention
